# The genome assembly of the farmed European whitefish *Coregonus lavaretus* L. from the Finnish selective breeding programme

**DOI:** 10.1186/s12863-025-01313-6

**Published:** 2025-03-25

**Authors:** Kisun Pokharel, Daniel Fischer, Terhi Iso-Touru, Ilma Tapio, Miika Tapio, Tuomas Leinonen, Antti Kause

**Affiliations:** 1 Natural Resources Institute Finland (Luke), Tietotie 4, Jokioinen, 31600 Finland; 2 Natural Resources Institute Finland (Luke), Latokartanonkaari 9, Helsinki, 00790 Finland

**Keywords:** Pacbio, OmniC, Assembly, European whitefish, Genome, Coregonus

## Abstract

**Objectives:**

European whitefish (*Coregonus lavaretus L*.) is a freshwater salmonid that inhabits cold regions of central and north Europe and Siberia. It is an important aquaculture species in Finland, selectively bred since 1999. The breeding programme has applied genomic selection that uses SNP markers and phenotypic data to improve traits such as growth, product quality, and fish health. Salmonids are known for chromosomal rearrangements, and the current *C. lavaretus* reference genome that is based on an individual from Switzerland may deviate from the Finnish one. Therefore, we have assembled a genome for the Finnish European whitefish. This allows us to better assess the genetic basis of fish traits and to enhance the accuracy of genomic selection.

**Data description:**

The genome of European whitefish was sequenced using a combination of Illumina and PacBio technologies and assembled using wtbg2 and HiRise software. The assembly has a size of 2.94 Gb and comprises 6,706 scaffolds with the scaffold N50 of 1.36 Mb.

## Objective

Modern breeding programmes are based on genomic selection in which the genetic superiority of individuals is estimated using thousands of DNA markers and phenotypic data on traits. Such data can be used to study the structure of a genome and genomic determination of traits using genome-wide association studies, for which a reference genome is needed.

*Coregonus* is a diverse circumpolar genus of *Salmonidae* that has been used to study adaption, genome duplication and its re-diploidisation, and genetic determination of fish characteristics [2, 6, 7, 9, 14]. European whitefish, *Coregonus lavaretus* L., is also farmed for human consumption. In Finland, it is the second most important farmed fish species with 0.6–1 million kg farmed annually. A national selective breeding programme has been ongoing since 1999 to improve production, quality and health traits [3, 10, 11, 17].

Single nucleotide polymorphisms (SNPs) have been identified by genotyping-by-sequencing [8] and used to estimate genomic breeding values, genetic variation and quantitative trait loci (QTLs) for growth and resistance against *Saprolegnia* fungus in the national breeding programme [3]. To assign the markers to chromosomes, the only available draft genome of the European whitefish sampled in Switzerland was used [6, 7]. European whitefish genome is highly diverse across populations due to historical isolation and adaptation to different ecological niches [6, 14]. Therefore, the Swiss Alpine whitefish genome might not accurately represent the Finnish population due to the distinct evolutionary paths post-glaciation, potentially leading to variations across populations in gene content, repeat elements, and structural variations [5].

In this study, we generated a genome assembly specifically for the Finnish whitefish breeding programme. The draft genome will play an important role in improving the accuracy of selective breeding and the analysis of genetic determination of fish traits. Moreover, sequencing of several fish across the *Coregonus* species complex will enable comparing genomes in the future.

## Data description

One mature female fish from the year class of 2019 was sampled from the Finnish national breeding programme maintained by Luke (Natural Resources Institute Finland) at the Enonkoski research station. This anadromous strain was originally collected in the late 1990’s from the Kokemäenjoki river (N 61° 32,757’; E 21° 42,951’). Since then, the cultivated stock has been kept closed without supplementing with new fish from the wild for four generations.

Tissue homogenates were prepared using liver (25 mg), muscle (26 mg) and fin (23 mg) tissues, followed by processing with the Nanobind kit (PacBio) to extract high molecular weight DNA. The liver sample was choosen for sequencing due to its higher DNA yield compared to other two tissues. The extracted liver DNA was quantified, cleaned, and sheared to 14–16 kb fragments for the preparation of two PacBio libraries. Six SMRTcells from these libraries were sequenced on the PacBio Sequel II, generating approximately 152.8 Gb of raw data. We used BLAST v2.9.0 [13] and in-house scripts to remove remnant barcodes and PacBio adapter sequences. Additionally, long subread data (15 Kb, 25 Kb, 40 Kb) was created by trimming 25 bp from both ends, and removing barcode and adapter sequences using cutadapt v4.1 [12] and blast v2.9.0, respectively. Omni-C libraries were prepared separately using the DNA extracted from fin, muscle and liver samples, following the Dovetail Omni-C kit Non-mammalian Sample Protocol version 1.2B – Animal Tissues (Cantata Bio). These libraries were sequenced on an Illumina NovaSeq 6000 instrument using paired-end strategy (NovaSeq S4 300 cycle partial lane with 2 × 150 bp reads) producing 357 GB of raw data. We removed 20 bp from the 5’ end of both reads using cutadapt v4.1, resulting in 310 Gb of clean Omni-C data.

We used hifiasm v0.19.5-r592 [4] to generate two genome assemblies. The main assembly (Table 1, Data file 1) includes both PacBio and OmniC sequence data, while an alternative assembly is based on PacBio sequence data only. The main assembly has a size of 2,940,286,296 bp and consists of 6,706 scaffolds, with an average scaffold length of 438,456 bp and the largest scaffold being 15,615,918 bp. The scaffold N50 and N90 values are 1,358,293 bp and 158,098 bp, respectively (Table 1, Data file 2). Similarly, the BUSCO (Benchmarking Universal Single Copy Orthologs) evaluation [19] using eukaryote database (eukaryote_odb10, *n* = 255) indicated 98.8% (*n* = 252) of complete BUSCOs with 2 fragmented and 1 missing BUSCOs. Out of 252 complete BUSCOs 164 were duplicated indicating heterozygosity of the assembly. The alternative assembly is slightly shorter but overall statistics are similar to the main assembly (Table 1, Data file 2). The genome assembly is publicly available in European Nucleotide Archive (ENA) at http://identifiers.org/ena.embl:PRJEB69218 [15]. Ploidy assessment using smudgeplot v0.4.0 [18] on PacBio Hi-Fi data indicated that 63% of the European whitefish genome is tetraploid or highly similar diploid paralogs for which genotyping can produce a maximum of four alleles (Table 1, Data file 3). The whole genome of the ancestor of salmonids was duplicated via autopolyploidization ~ 80–100 million years ago, after which the genome has undergone a re-diploidization process which is not completed in European whitefish.

**Table 1:** Overview of data files

Label	Name of data file	File types (file extension)	Data repository and identifier (DOI or accession number)
Data file 1	Genome assembly of European whitefish from Finland	Fasta file (fasta.gz)	European Nucletotide Archive (http://identifiers.org/ena.embl:PRJEB69218) [15]
Data file 2	Summary of genome assemblies	Portable document format (.pdf)	Figshare (10.6084/m9.figshare.28284377.v11 ) [16]
Data file 3	Smudgeplot of European whitefish genome	Portable document format(.pdf)	Figshare (10.6084/m9.figshare.28284377.v11) [16]

## Limitations

One of the main limitations of our study is that the current assembly is at the scaffold level, and additional experiments are required to obtain karyotype information to assign scaffolds to chromosomes. Additionally, the assembly lacks genome annotation, which is critical for identifying gene locations, regulatory elements, and functional genomic regions. Furthermore, the inclusion of Omni-C libraries did not improve the quality of the assembly. The genome of European whitefish is more complex than other diploid species due to the presence of several duplicated regions [3, 7], which adds an extra layer of difficulty to achieving a high-quality assembly. These duplications complicate the assembly process, as the accurate phasing of duplicated regions is challenging with current technologies. This may indicate the need for further optimization of long-read sequencing, and the use of additional complementary methods, such as phased assembly techniques or haplotype-resolved sequencing, to fully resolve these complex regions and produce a more complete and reliable genome assembly [1].

## Data Availability

The main assembly that includes both PacBio and Omni-C data is publicly available in ENA at http://identifiers.org/ena.embl:PRJEB69218 [15] and the alternative assembly that excludes Omni-C data is available upon request from Antti Kause. Data files 2 and 3 are available at 10.6084/m9.figshare.28284377.v11 [16].

## References

[1] Abou Saada O, Tsouris A, Eberlein C, Friedrich A, Schacherer J. nPhase: an accurate and contiguous phasing method for polyploids. Genome Biol. 2021;22:126. 10.1186/s13059-021-02342-x. 33926549 10.1186/s13059-021-02342-x PMC8082856

[2] Borovikova EA, Malina JI. Phylogeography of common Whitefish (Coregonus Lavaretus L.) of Northwestern Russia. Contemp Probl Ecol. 2018;11:286–96. 10.1134/S1995425518030058.

[3] Calboli FCF, Iso-Touru T, Bitz O, Fischer D, Nousiainen A, Koskinen H, et al. Genomic selection for survival under naturally occurring saprolegnia oomycete infection in farmed European Whitefish Coregonus Lavaretus. J Anim Sci. 2023;101:skad333. 10.1093/jas/skad333. 37777972 10.1093/jas/skad333 PMC10583997

[4] Cheng H, Concepcion GT, Feng X, Zhang H, Li H. Haplotype-resolved de Novo assembly using phased assembly graphs with hifiasm. Nat Methods. 2021;18:170–5. 10.1038/s41592-020-01056-5. 33526886 10.1038/s41592-020-01056-5 PMC7961889

[5] Crotti M, Bean CW, Gowans ARD, Winfield IJ, Butowska M, Wanzenböck J, et al. Complex and divergent histories gave rise to genome-wide divergence patterns amongst European Whitefish (Coregonus lavaretus). J Evol Biol. 2021;34:1954–69. 10.1111/jeb.13948. 34653264 10.1111/jeb.13948 PMC9251650

[6] De-Kayne R, Selz OM, Marques DA, Frei D, Seehausen O, Feulner PGD. Genomic architecture of adaptive radiation and hybridization in alpine Whitefish. Nat Commun. 2022;13:4479. 10.1038/s41467-022-32181-8. 35918341 10.1038/s41467-022-32181-8 PMC9345977

[7] De-Kayne R, Zoller S, Feulner PGD. A de Novo chromosome-level genome assembly of Coregonus Sp. Balchen: one representative of the Swiss alpine Whitefish radiation. Mol Ecol Resour. 2020;20:1093–109. 10.1111/1755-0998.13187. 32395896 10.1111/1755-0998.13187 PMC7497118

[8] Fischer D, Tapio M, Bitz O, Iso-Touru T, Kause A, Tapio I. Fine-tuning GBS data with comparison of reference and mock genome approaches for advancing genomic selection in less studied farmed species. BMC Genomics. 2025;26:111. 10.1186/s12864-025-11296-4. 10.1186/s12864-025-11296-4 PMC11796084 39910437

[9] Hebert FO, Renaut S, Bernatchez L. Targeted sequence capture and resequencing implies a predominant role of regulatory regions in the divergence of a sympatric lake Whitefish species pair (Coregonus clupeaformis). Mol Ecol. 2013;22:4896–914. 10.1111/mec.12447. 23962219 10.1111/mec.12447

[10] Kause A, Quinton C, Airaksinen S, Ruohonen K, Koskela J. Quality and production trait genetics of farmed European Whitefish, Coregonus Lavaretus. J Anim Sci. 2011;89:959–71. 10.2527/jas.2010-2981. 21097679 10.2527/jas.2010-2981

[11] Kause A, Quinton CD, Ruohonen K, Koskela J. Genetic potential for the regulation of variability in body lipid and protein content of European Whitefish (Coregonus lavaretus). Br J Nutr. 2009;101:1444–51. 10.1017/S0007114508091265. 18826727 10.1017/S0007114508091265

[12] Krueger F. A wrapper around Cutadapt and FastQC to consistently apply adapter and quality trimming to FastQ files, with extra functionality for RRBS data: FelixKrueger/TrimGalore. 2019. Available at: https://github.com/FelixKrueger/TrimGalore. Accessed 4 Sept 2019.

[13] Madden T. The BLAST Sequence Analysis Tool. National Center for Biotechnology Information (US). 2003. Available at: https://www.ncbi.nlm.nih.gov/books/NBK21097/. Accessed 4 Sept 2019.

[14] Mérot C, Stenløkk KSR, Venney C, Laporte M, Moser M, Normandeau E, et al. Genome assembly, structural variants, and genetic differentiation between lake Whitefish young species pairs (Coregonus sp.) with long and short reads. Mol Ecol. 2023;32:1458–77. 10.1111/mec.16468. 35416336 10.1111/mec.16468

[15] Pokharel K, Fischer D, Iso-Touru T, Tapio I, Tapio M, Leinonen T, Kause A. Siika: European whitefish (*Coregonus lavaretus*) genome from Finland. ENA. 2024. http://identifiers.org/ena.embl:PRJEB69218. 10.1186/s12863-025-01313-6 PMC11934727 40128681

[16] Pokharel K, Fischer D, Iso-Touru T, Tapio I, Tapio M, Leinonen T, Kause A. Data files: reference genome of European Whitefish (Coregonus Lavaretus L.) from Finland. Figshare. 2025. 10.6084/m9.Figshare.28284377.v11. 10.1186/s12863-025-01313-6 PMC11934727 40128681

[17] Quinton CD, Kause A, Ruohonen K, Koskela J. Genetic relationships of body composition and feed utilization traits in European Whitefish (Coregonus Lavaretus L.) and implications for selective breeding in fishmeal- and soybean meal-based diet environments. J Anim Sci. 2007;85:3198–208. 10.2527/jas.2006-792. 17709787 10.2527/jas.2006-792

[18] Ranallo-Benavidez TR, Jaron KS, Schatz MC. GenomeScope 2.0 and smudgeplot for reference-free profiling of polyploid genomes. Nat Commun. 2020;11:1432. 10.1038/s41467-020-14998-3. 32188846 10.1038/s41467-020-14998-3 PMC7080791

[19] Simão FA, Waterhouse RM, Ioannidis P, Kriventseva EV, Zdobnov EM. BUSCO: assessing genome assembly and annotation completeness with single-copy orthologs. Bioinformatics. 2015;31:3210–2. 10.1093/bioinformatics/btv351. 26059717 10.1093/bioinformatics/btv351

